# Infiltrative Basal Cell Carcinoma of the Head: Factors Influencing Bone Invasion and Surgical Outcomes

**DOI:** 10.3390/life15040551

**Published:** 2025-03-28

**Authors:** Anđela Dimkić Milenković, Vladimir Milenković, Masa Petrovic, Ana Tomic, Aleksandar Matejic, Nina Brkic, Milan Jovanović

**Affiliations:** 1 University Clinical Center of Serbia, Pasterova 2, 11000 Belgrade, Serbia; 2 Faculty of Medicine, University of Belgrade, 11000 Belgrade, Serbia; 3 Institute for Cardiovascular Diseases “Dedinje”, Heroja Milana Tepica 1, 11000 Belgrade, Serbia; 4 Department of Plastic and Reconstructive Surgery, Institute for Orthopedic Surgery “Banjica”, 11000 Belgrade, Serbia

**Keywords:** basal cell carcinoma, bone invasion, infiltrative, risk factors, surgery

## Abstract

Basal cell carcinoma (BCC) is the most prevalent form of skin cancer, with infiltrative subtypes presenting significant clinical challenges due to their aggressive behavior and potential for deep tissue and bone invasion. This study aimed to evaluate factors associated with bone infiltration and surgical bone removal in patients with infiltrative BCC. A prospective cohort of 100 patients with histologically confirmed infiltrative BCC was analyzed retrospectively. Clinical, histopathological, and imaging data were assessed to identify predictors of tumor behavior. Tumor size, histological subtype, and disease duration emerged as significant factors associated with bone invasion and resection. Logistic regression analysis identified tumor length as the most significant predictor of both bone infiltration (OR = 1.102, *p* < 0.001) and surgical bone removal (OR = 1.105, *p* < 0.001). Aggressive subtypes, such as infiltrative and morpheaform BCC, were more likely to invade bone and exhibited higher recurrence rates. While these findings highlight the importance of early detection and individualized treatment strategies, the absence of data on sun exposure and urban-rural residency limits the broader applicability of the results. Future research addressing these variables will provide a more comprehensive understanding of BCC aggressiveness and improve clinical management of this malignancy.

## 1. Introduction

Basal cell carcinoma (BCC) is the most common form of skin cancer, comprising approximately 80% of non-melanoma skin cancers globally and affecting millions annually [1]. Arising primarily from the basal cells of the epidermis, BCC has a unique clinical profile. While it rarely metastasizes, it can cause significant local morbidity due to its destructive capacity [2]. The incidence of BCC is higher in fair-skinned individuals and populations residing in regions with high ultraviolet (UV) radiation exposure, including parts of Australia, Europe, and the United States [3]. Chronic UV exposure remains a leading etiological factor for BCC, contributing to DNA damage and genetic mutations that initiate oncogenic pathways [4]. Among these pathways, the hedgehog signaling pathway, often disrupted in BCC through mutations in the PTCH1 gene, is considered central to the pathogenesis of this malignancy [5,6].

Histologically, BCC is a heterogeneous entity, encompassing several subtypes with varying growth patterns, aggressiveness, and recurrence rates. While the nodular and superficial subtypes are the most frequently observed and are often amenable to standard excision, other subtypes such as infiltrative, micronodular, and sclerosing BCC exhibit more aggressive behavior [5]. The infiltrative subtype, in particular, is marked by narrow, irregular strands of basaloid cells that penetrate deeply into the dermis and subcutaneous tissue, often extending beyond clinically visible margins [7]. This infiltrative growth pattern is associated with a significantly higher risk of incomplete excision and subsequent recurrence, and in severe cases, it may invade surrounding muscle, cartilage, and even bone, leading to considerable disfigurement and functional impairment [8].

The clinical management of BCC, especially the infiltrative subtype, necessitates careful consideration of both surgical and non-surgical treatment modalities. Surgical excision, including Mohs micrographic surgery (MMS), is the primary treatment for infiltrative BCC and provides the highest cure rates due to comprehensive examination of excised tissue margins [9]. However, even with MMS, the diffuse and aggressive nature of infiltrative BCC can complicate treatment, necessitating wider surgical margins and, at times, multiple stages to ensure complete tumor clearance [9]. Non-surgical therapies, such as radiation and hedgehog pathway inhibitors like vismodegib and sonidegib, have been explored for advanced or inoperable BCC cases, offering alternative approaches for patients who may not be ideal candidates for extensive surgery [8]. Nonetheless, the infiltrative subtype continues to pose a unique challenge due to its high propensity for local invasion and recurrence.

Understanding the factors that contribute to the aggressive behavior of infiltrative BCC is crucial for improving treatment outcomes. It has been previously noted that there are several risk factors for deeper tissue invasion, including tumor size, location (particularly in high-risk areas like the head and neck), and histological subtype, with infiltrative and sclerosing patterns associated with a higher risk of bone invasion. These factors are essential for developing individualized treatment plans and determining appropriate follow-up intervals. Additionally, genetic studies have highlighted the role of specific mutations, particularly in the hedgehog pathway, as drivers of BCC pathogenesis. Mutations in PTCH1, SMO, and other components of this pathway have been linked to both increased cellular proliferation and resistance to certain therapies, making them targets of interest for future therapeutic development [5,6].

The primary aim of this study is to identify and analyze the factors contributing to bone infiltration in infiltrative BCC cases, with secondary outcomes focused on determining risk factors associated with bone resection. This study seeks to evaluate the aggressiveness of sporadic infiltrative BCC and to provide insights into both risk stratification and management strategies aimed at reducing recurrence and improving surgical outcomes.

## 2. Materials and Methods

### 2.1. Study Design

This study was designed as a prospective cohort with retrospective analysis, conducted at the University Clinical Center of Serbia, Clinic for Burns, Plastic and Reconstructive Surgery.

### 2.2. Participants

The initial cohort included 103 consecutive patients over the age of 18 who presented with basal cell carcinoma (BCC) between November 2017 and May 2019. After applying exclusion criteria, which removed three patients with multiple lesions, Gorlin-Goltz syndrome, or xeroderma pigmentosum, the final study population comprised 100 participants. The inclusion criteria required patients to be over 18 years of age with a confirmed diagnosis of infiltrative BCC localized in the head (skull) regions (frontal, parietal, temporal, or occipital) who underwent surgical treatment. Exclusion criteria were implemented to maintain a focus on isolated, non-syndromic cases of BCC, thereby excluding individuals with known genetic predispositions or multiple lesions.

### 2.3. Data Collection

All participants underwent a comprehensive clinical examination to assess the characteristics and invasiveness of their BCC lesions. For tumors that were both fixated and adherent upon physical examination, additional diagnostic imaging, specifically computed tomography (CT), was performed to evaluate the extent of local invasion and potential bone involvement. These diagnostic findings were then retrospectively analyzed to identify factors associated with bone infiltration and to determine risk factors that could necessitate bone resection.

In the study, tumor length was defined as the longest diameter of the lesion measured on the skin surface, while tumor width was defined as the shortest perpendicular diameter to the tumor length. Tumor thickness was defined as the longest diameter from tumor outer surface to deep structures. Tumor length and width were measured by the surgeon using a standard medical ruler, expressed in millimeters (mm). The length was recorded first, followed by the width, ensuring consistency. Measurements were performed preoperatively by the same examiner to minimize interobserver variability. After surgical removal of the lesion as a whole, the specimen was sent to the pathology department where a pathologist measured the tumor thickness and classified the tumor. BCC subtypes were classified based on histopathological reports. In cases where mixed patterns were observed, the pathologist reported the subtypes.

### 2.4. Ethical Consideration

Ethical approval for this study was obtained from the Ethics Committee of the University of Belgrade, Faculty of Medicine (260/IV-19), on 10 April 2018.

### 2.5. Statistical Analysis

Results are presented as count (%), mean ± standard deviation. or median (interquartile (IQ) range: 25th–75th Percentile), depending on data type and distribution. Groups are compared using parametric (*t* test) and nonparametric (Chi-square, Mann–Whitney U test) tests. Logistic regression was performed to evaluate the relationship between dependent variable and independent variables. All *p* values less than 0.05 were considered significant. All data were analyzed using SPSS 29.0 (IBM Corp. Released 2020. IBM SPSS Statistics for Windows, Version 29.0. Armonk, NY, USA: IBM Corp.).

## 3. Results

Among 100 participants, 14 (14%) were diagnosed with BCC that exhibited bone invasion. Participants in the bone invasion group tended to be slightly younger, with a median age of 69 years (range: 51–90 years), compared to those without bone invasion, who had a median age of 72.5 years (range: 33–91 years). Despite this observed age difference, the variation was not statistically significant (U = 495.0, *p* = 0.287). The group with bone invasion included nine (64.3%) male participants, whereas the group without bone invasion comprised 43 (50.0%) male participants. However, this difference in gender distribution also lacked statistical significance (χ^2^ = 0.984, *p* = 0.321).

Detailed tumor clinical characteristics are summarized in Table 1, where it is interesting to note that the rate of bone invasion didn’t significantly differ based on the tumor thickness (*p* = 0.389).

**Figure 1:**
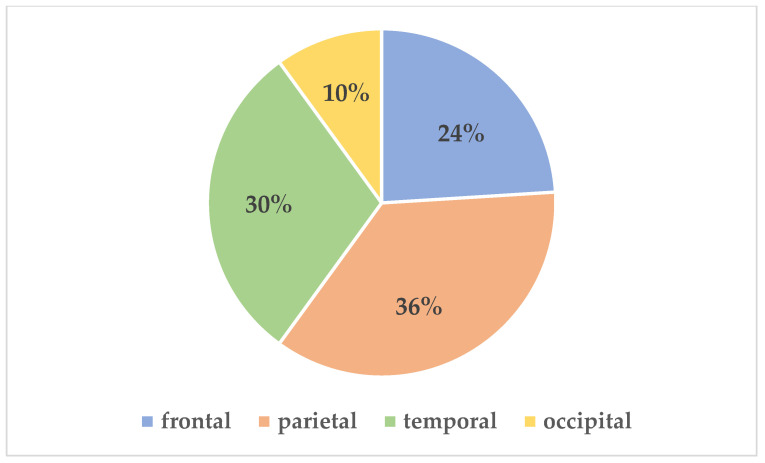
Distribution of Basal Cell Carcinoma by Anatomical Location.

Tumors with bone invasion were predominantly located in the parietal and temporal regions of the scalp (*p* = 0.018) and typically demonstrated clinically indolent behavior (*p* = 0.003) (Figure 1).

**Figure 2:**
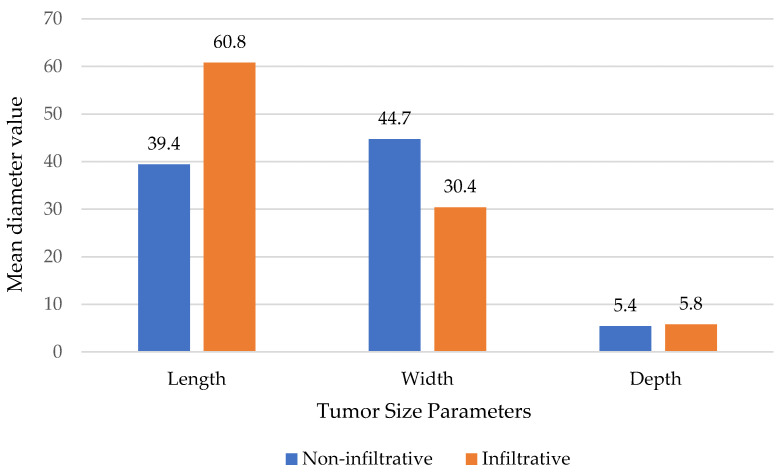
Distribution of Different Tumor Diameters (Length, Width, and Depth) and Major Histopathological Subtypes of Basal Cell Carcinoma.

These lesions were present for an extended period, often approximately five years, before diagnosis or intervention (*p* = 0.001). Importantly, tumors with bone invasion were significantly larger in both their longitudinal and transverse diameters (*p* < 0.001) (Figure 2).

From a histopathological perspective, bone-invading tumors were primarily classified as nodular or nodular-infiltrating types (*p* = 0.007). It is noteworthy that all morphoeic variants observed in the study exhibited loco-regional bone invasion.

A logistic regression model for bone invasion risk prediction is presented in Table 2. Our analysis identified tumor length as the only significant predictor of bone invasion, with an odds ratio (OR) of 1.102 (95% CI: 1.044–1.165, *p* < 0.001). Localization, tumor width, and histopathologically confirmed infiltrative types were not identified as significant predictors of tumor-related bone invasion.

Out of all noted participants, 18 (18%) underwent surgical bone excision as part of their treatment. These individuals were slightly older, with a median age of 73 years (range: 51–90 years), compared to those whose bone was preserved. However, this age difference was not statistically significant (U = 965.5, *p* = 0.703). Among participants undergoing bone removal, 11 were male, and this gender distribution did not differ significantly from the group with surgically preserved bone tissue (χ^2^ = 0.984, *p* = 0.321).

Tumor and clinical characteristics in relation to surgical bone removal are outlined in Table 3. Similar to tumors associated with bone invasion, those requiring bone excision were predominantly located in the parietal and temporal regions (*p* = 0.004), exhibited indolent clinical behavior (*p* = 0.015), and had been present for approximately five years (*p* < 0.001). These tumors were significantly larger in both length and width compared to tumors in participants without bone removal (*p* < 0.001), and exhibited nodular or nodular-infiltrating histological patterns (*p* = 0.010). However, the rate of surgical bone removal didn’t significantly differ based on the tumor thickness (*p* = 0.801).

The logistic regression model for surgical bone removal prediction is presented in Table 4. Our analysis identified tumor length as the sole significant predictor for surgical bone removal, with an OR of 1.105 (95% CI: 1.053–1.160, *p* < 0.001). Localization, tumor width, and histopathologically confirmed infiltrative types were not identified as significant predictors of surgical bone removal.

## 4. Discussion

The purpose of this study was to gain insights into the behavior and treatment of the various histopathological types of basal cell carcinoma (BCC), including their propensity for bone invasion. Prospective data were analyzed from 103 patients at the Clinic for Burns, Plastic and Reconstructive Surgery in the University Clinical Centre of Serbia, Belgrade.

Bone invasion by BCC is an uncommon occurrence, typically associated with neglected, long-standing tumors or those in anatomically high-risk areas, such as the scalp. It can significantly complicate treatment, often necessitating more extensive surgical resections and, in some cases, reconstruction [10]. This aggressive behavior is generally more common in patients with large, recurrent, or neglected lesions.

Consistent with the literature, our cases involved BCCs that had been present for several years before treatment, underlining the critical importance of early detection [11]. In accordance with the results of other studies done in a similar field, patients in our study were predominantly male [12,13,14]. In the study of Rahimi-Nedjat et al. [12], the mean patient age was 77.1 years, while the mean ages of patients in studies conducted by Rudolph C et al. [13] and Stang A. et al. [14] were 70 and 65 years, respectively. Similar to these studies, the mean age in our study was 72 years, which suggests that a routine visit to a dermatologist at this age is highly recommended. Some authors suggest this might be because of hair length and androgenic alopecia in male patients, which makes their scalp more susceptible to UV radiation, which is a risk factor [15].

Unlike the study by Pyne et al. in Australia, which analyzed 4565 cases and found a significant link between male sex and tumor infiltration, our study found no correlation between sex and the occurrence of bone invasion [16].

Basal cell carcinoma of the scalp prevalence varies in the literature from 1.1–2.7% up to even 29.2% [17,18,19].

Histopathologically, BCCs that invade bone often exhibit more aggressive subtypes, such as infiltrative, morpheaform, or basosquamous variants. Also, some subtypes are more common in certain locations [20]. Moreover, our results show that the histological type of tumor is a statistically significant predictor of bone invasion, as are tumor width and length. In our study, the most common histological type in a group of patients with bone invasion was infiltrative, while in the other group it was nodular-infiltrative. This contrasts with some other studies, which included patients with BCC of the scalp and found nodular histological type to be the most common [18,21,22]. The reason for this might be because they included patients with a history of radiotherapy of the scalp (tinea capitis), which is recognized as one of the main risk factors. However, Tessone et al. in their 2012 study didn’t find genetic differences between scalps of irradiated patients and nonirradiated patients [23].

The rate of tumor recurrence of 22% in group with patients with bone invasion and 8.5% in the group without bone invasion, which is coherent with results found in the literature [24]. Our findings highlight the importance of a careful diagnostic evaluation of all BCC patients, particularly those at risk of bone invasion, as this can significantly impact treatment planning and prognosis.

The study has several limitations that should be acknowledged. First, due to the predefined data collection protocols that were retrospectively analyzed, we did not include detailed records on occupational or recreational sun exposure, as well as differences in environmental exposure and healthcare access between urban and rural populations, which is a known risk factor for keratinocyte cancers, including basal cell carcinoma (BCC). Previous research has demonstrated a significant association between cumulative sun exposure and the development of BCC, emphasizing the importance of exposure measurement in studies of this nature [25,26]. The absence of detailed exposure data limits our ability to evaluate the potential interaction between environmental and genetic factors in our cohort.

Second, the population division based on urban and rural residency was not explicitly accounted for. Given that environmental exposure and access to healthcare services differ between urban and rural areas, the lack of stratification may have influenced the generalizability of our findings. Urban populations may have different patterns of sun exposure due to occupational and recreational activities compared to rural populations, where outdoor work is more prevalent. Additionally, while limiting our study to BCC of the head enhances anatomical consistency and clinical relevance, particularly regarding the risk of bone invasion, we acknowledge that this approach may limit the generalizability of our findings for BCC in other anatomical locations

Furthermore, the exclusion of patients with multiple lesions, Gorlin-Goltz syndrome, or xeroderma pigmentosum represents a limitation of our study, as it prevents us from assessing the influence of these genetic syndromes on BCC behavior and bone invasion. Future research including these patient groups could provide a more comprehensive understanding of these factors.

Another limitation of our study is the potential discrepancy between surgical decision-making and histopathological confirmation of bone invasion. While bone resection was performed in cases where the tumor affected the periosteum, this does not always correspond to true bone invasion as defined by histopathological criteria. As a result, the overlap between cases of bone resection and confirmed bone invasion may not be exact, but it remains relatively close, likely due to the small sample size. This similarity may have led to comparable statistical analysis results when comparing the surgical and histopathological groups.

Future research addressing these limitations—particularly through the incorporation of standardized data collection of the aforementioned socioeconomic and geographic factors, sun exposure metrics, genetic syndrome, and urban-rural stratification —will be critical to furthering our understanding of the epidemiology and outcomes of keratinocyte cancers. Additionally, future studies should be conducted with larger cohorts, standardized criteria for surgical bone resection, which may help further clarify these findings, and the inclusion of the tumor thickness to subcutaneous fat thickness ratio.

## 5. Conclusions

This study highlights the significant challenges associated with infiltrative BCC, particularly its potential for aggressive behavior and bone invasion. The findings indicate that the invasiveness and propensity for deeper tissue infiltration in BCC are strongly influenced by the histological type and tumor size. Larger tumors and aggressive subtypes, such as infiltrative and morpheaform variants, were shown to have a pronounced association with bone infiltration and the requirement for surgical bone removal. These observations emphasize the critical importance of prompt diagnosis and individualized therapeutic approaches to minimize the risks of advanced invasion and recurrence.

## Figures and Tables

**Table 1:** Tumor characteristics based on the bone invasion.

	Bone Invasion	No Bone Invasion	
	N = 14 (14%)	N = 86 (86%)	*p* *
**Tumor localization**			*p* = 0.018
Frontal	0 (0%)	24 (100%)	
Parietal	9 (25.0%)	27 (75.0%)	
Occipital	0 (0%)	10 (100%)	
Temporal	5 (16.7%)	25 (83.3%)	
**Tumor length (mm)**	100 (30–140)	22 (10–67)	*p* < 0.001
**Tumor width (mm)**	70 (18–110)	18 (10–59)	*p* < 0.001
**Tumor thickness (mm)**	2 (0.5–12)	3 (0.5–12)	*p* = 0.325
**Disease duration**			*p* = 0.001
1 year	0 (%)	24 (100%)	
2 years	0 (%)	45 (100%)	
3 years	1 (5.6%)	17 (94.4%)	
5 years	13 (100%)	0 (0%)	
**Clinical indolence**			*p* = 0.003
Indolent tumor	0 (0%)	35 (100%)	
Non indolent tumor	14 (21.5%)	51 (78.5%)	
**Histopathological finding**			*p* = 0.007
Superficial	0 (0%)	4 (100%)	
Nodular	0 (0%)	21 (100%)	
Nodular-infiltrative	3 (10.7%)	25 (89.3%)	
Infiltrative	7 (25.9%)	20 (74.1%)	
Nodular cystic variant	0 (0%)	9 (100%)	
Adenoid	0 (0%)	1 (100%)	
Micronodular	0 (0%)	2 (100%)	
Morpheaform (morphoeic)	2 (100%)	0 (0%)	
Basosquamous	2 (33.3%)	4 (66.7%)	
**Resection lines**			*p* = 0.458
Clean/No tumor tissue	13 (15.3%)	71 (84.7%)	
Lined with tumor tissue	1 (6.7%)	14 (93.3%)	
**Tumor reoccurrence**			*p* = 0.182
Yes	3 (27.3%)	8 (72.7%)	
No	11 (12.4%)	78 (87.6%)	

* All *p* values less than 0.05 were considered significant; mm—millimeters.

**Table 2:** Logistic regression analysis for bone invasion risk.

	Univariate Regression Analysis		Multivariate Regression Analysis	
	OR (CI)	*p* Value	OR (CI)	*p* Value
Localization	0.810 (0.250–2.621)	0.724		
Length	1.109 (1.049–1.171)	<0.001	1.102 (1.044–1.165)	<0.001
Width	1.110 (1.059–1.164)	<0.001		
HP (infiltrative type)	2.278 (0.0663–7.827)	0.191		

OR—Odds Ratio; CI—Confidence Interval.

**Table 3:** Tumor characteristics based on the bone removal.

	Bone Removed	No Bone Removal	
	N = 18 (18%)	N = 82 (82%)	*p* *
**Tumor localization**			*p* = 0.004
Frontal	0 (0%)	25 (100%)	
Parietal	11 (30.6%)	25 (69.4%)	
Occipital	0 (0%)	9 (100%)	
Temporal	7 (23.3%)	23 (76.7%)	
**Tumor length (mm)**	80 (20–140)	22 (10–63)	*p* < 0.001
**Tumor width (mm)**	65 (14–110)	17.5 (10–59)	*p* < 0.001
**Tumor thickness (mm)**	2 (0.5–12)	3 (0.5–12)	*p* = 0.699
**Disease duration**			*p* < 0.001
1 year	0 (0%)	24 (100%)	
2 years	0 (0%)	45 (100%)	
3 years	5 (27.8%)	13 (72.2%)	
5 years	13 (100%)	0 (0%)	
**Clinical indolence**			*p* = 0.015
Indolent tumor	0 (0%)	35 (100%)	
Non indolent tumor	18 (27.7%)	47 (72.3%)	
**Histopathological finding**			*p* = 0.01
Superficial	0 (0%)	4 (100%)	
Nodular	0 (0%)	21 (100%)	
Nodular-infiltrative	4 (14.3%)	24 (85.7%)	
Infiltrative	9 (33.3%)	18 (66.7%)	
Nodular cystic variant	0 (66.7%)	9 (33.3%)	
Adenoid	0 (0%)	1 (100%)	
Micronodular	1 (50%)	1 (50%)	
Morpheaform (morphoeic)	2 (100%)	0 (0%)	
Basosquamous	2 (33.3%)	4 (66.7%)	
**Resection lines**			*p* = 0.730
Clean/No tumor tissue	16 (18.8%)	69 (81.2%)	
Lined with tumor tissue	2 (13.3%)	13 (86.7%)	
**Tumor reoccurrence**			*p* = 0.107
Yes	4 (36.4%)	7 (63.3%)	
No	14 (15.7%)	75 (84.3%)	

* All *p* values less than 0.05 were considered significant; mm—millimeters.

**Table 4:** Logistic regression analysis for bone removal prediction.

	Univariate Regression Analysis		Multivariate Regression Analysis	
	OR (CI)	*p* Value	OR (CI)	*p* Value
Localization	0.945 (0.332–2.688)	0.915		
Length	1.427 (1.113–1.830)	0.005	1.105 (1.053–1.160)	<0.001
Width	1.109 (1.050–1.172)	<0.001		
HP (infiltrative component)	2.476 (0.809–7.579)	0.112		

OR—Odds Ratio; CI—Confidence Interval.

## Data Availability

The data presented in this study are available upon request from the corresponding author due to privacy reasons.

## Abbreviations

The following abbreviations are used in this manuscript:

BCC	Basal cell carcinoma
UV	Ultraviolet
MMS	Mohs micrographic surgery
OR	Odds Ratio
CI	Confidence Interval
